# Comparison of Psychometric Characteristics for Five Versions of the Interpersonal Needs Questionnaire in Teenagers Sample

**DOI:** 10.3389/fpsyg.2021.676361

**Published:** 2021-05-28

**Authors:** Jiaxin Quan, Xiaofang Yu, Yan Cai, Dongbo Tu

**Affiliations:** ^1^School of Psychology, Jiangxi Normal University, Nanchang, China; ^2^Center of Mental Health Education and Research, School of Psychology, Jiangxi Normal University, Nanchang, China

**Keywords:** teenagers, item response theory, comparison, psychometric characteristics, interpersonal needs questionnaire

## Abstract

Interpersonal Needs Questionnaire (INQ) is a self-report measure of perceived burdensomeness and thwarted belongingness with five versions in recent studies. There are five versions of INQ. But results from studies using different versions are quite different. Current suicide behavior among teenagers has attracted much attention. But which version is more suitable for teenage samples is still uncertain. It is important to compare the potential differences in different versions of INQ to identify the most psychometrically available version to predict teenagers' acquired capability for suicide and provide them with timely help to reduce teenagers' suicide rates. This study compared the construct validity, internal consistency, validity, and average test information of each version in the sample of teenagers. Results showed the 10-item version provided the most average test information in both thwarted belongingness subscale and perceived burdensomeness subscale, and the INQ-10 is more suitable for teenage samples.

## Introduction

Suicide is a major social and public health problem facing the world. Nearly 800,000 people commit suicide each year, and the number of suicide attempts is many times the number of suicides. Suicide occurred throughout the lifespan and it was the third leading cause of death in 15–19-year-old worldwide in 2016 (World Health Organization, 2019). In the general population, attempted suicide is the biggest risk factor for suicide. Suicide attempts peak in mid-puberty (Carballo et al., 2019). Suicide and serious self-harm not only seriously endanger the lives and health of young people, but also cause serious losses to individuals, families, and society.

The interpersonal–psychological theory of suicide (IPTS) was first proposed by Joiner (2005) and further expanded by Van Orden et al. (2010). This theory surpasses the previous theories of suicide in that it explains why the vast majority of people with suicidal ideation do not attempt suicide. IPTS proposes that suicidal behavior occurs only when an individual has both the desire to die and the acquired capability to engage in suicidal behavior. The desire to die is an individual's desire to end her/his life, which roughly corresponds to the common definition of suicidal ideation (Van Orden et al., 2008a). The acquired ability to engage in suicidal behavior is a learned ability, which means that through repeated exposure to painful and provocative events, the fear of death can be reduced and the tolerance of physical pain can be enhanced. According to the prediction of IPTS (Joiner, 2005; Van Orden et al., 2010), whether an individual has suicidal ideation depends on whether belongingness of the individual is met (thwarted belongingness, TB) and whether the individual considers himself/herself a burden to others (perceived burdensomeness, PB), and suicidal ideation will not turn into suicidal behavior until the acquired capability is large enough. Therefore, the interpersonal–psychological theory of suicide is defined as the framework of *ideation-to-action* (Klonsky and May, 2014; Klonsky et al., 2016).

Since the interpersonal–psychological theory of suicide was proposed, it has inspired many empirical studies on the causes of suicidal ideation, attempts, and fatalities. Research on the interpersonal–psychological theory of suicide has been conducted in different samples, such as undergraduates (Hagan et al., 2015; Suh et al., 2017), prison inmates (Mandracchia and Smith, 2015), physicians (Fink-Miller, 2015), older adults (Cukrowicz et al., 2013), psychiatric inpatients and outpatients (Monteith et al., 2013), military service members (Bryan et al., 2010), sexual minorities (Silva et al., 2015), and firefighters (Chu et al., 2016). The interpersonal–psychological theory of suicide was also validated cross-culturally across Korean and US undergraduate students (Suh et al., 2017). Moreover, based on IPTS, Van Orden (2009) further confirmed and extended the theory that perceived burdensomeness and thwarted belongingness were combined into interpersonal needs and constructed a corresponding Interpersonal Needs Questionnaire (INQ) with 25 items to reflect whether current interpersonal relationship needs of the individual were met. On account of the multicollinearity between thwarted belongingness and perceived burdensomeness, the 12-item INQ was later developed (Van Orden et al., 2008a). And the original authors proposed a 15-item version (Van Orden et al., 2012). An 18-item version was validated in a book on the interpersonal theory (Joiner et al., 2009) and a 10-item INQ was validated for use in military samples (Bryan et al., 2010). Each of the shorter versions of INQ is a subset of the original 25-item version. The 18-item version has been used primarily in the older adult, veterans, and college student samples in the US and college student samples in China (e.g., Davidson et al., 2011; Rasmussen and Wingate, 2011; Wong et al., 2011; Monteith et al., 2013; Zhang et al., 2013; Suh et al., 2017). The 15-item version introduced as an empirically derived refinement of the INQ-25 has been used in college student samples in the US, Singapore, China, and Switzerland (INQ-15; e.g., Van Orden et al., 2012; Hill and Pettit, 2013; Li et al., 2015; Baertschi et al., 2017; Teo et al., 2018), and Hallensleben et al. (2016) administrated the INQ-15 to a sample of German general population aged 14–75 years. The 12-item version has been used in a variety of samples in the US (e.g., Van Orden et al., 2008a; Davidson et al., 2009; Freedenthal et al., 2011; Hill and Pettit, 2012; Lamis and Lester, 2012). The 10-item version has been used primarily in military samples (e.g., Bryan et al., 2010, 2012, 2013; Bryan, 2011). According to previous studies (e.g., Bryan, 2011; Davidson et al., 2011; Freedenthal et al., 2011; Baertschi et al., 2017), the original version and its four shorter versions had acceptable internal consistencies and validities.

Although previous studies showed the INQ predicted suicide behaviors significantly (e.g., Van Orden et al., 2008a,b), a large number of discrepant studies found different versions of INQ had some differences in predicting suicide behaviors. For example, the INQ-25 (Anestis and Joiner, 2011), the INQ-18 (Wong et al., 2011), the INQ-12 (Hill and Pettit, 2012), and INQ-10 (Bryan et al., 2012) have been confirmed that perceived burdensomeness was a significant predictor, but thwarted belongingness was not. On the contrary, both perceived burdensomeness and thwarted belongingness had adequate predictive validity in the 12-item version (Lamis and Malone, 2011) and the 15-item version (Van Orden et al., 2012). The differences in the predictive validity of the measures confuse future researchers on which version of INQ should be used. Furthermore, there is documentation on how to select items in the previous literature for the INQ-25 and INQ-15. But for the other versions, it is unclear how to select items from the original 25-item. Therefore, it is necessary to investigate and compare the psychometric characteristics of the five versions of the INQ in the same sample.

In the past, most psychological constructs of self-rating measurements have been assessed through classical test theory (CTT), which focuses on construct validity, internal consistency, and test-retest stability (Hunsley and Mash, 2008). However, CTT methods to assess interpersonal needs of an individual rely on the total score or transformed total score and fail to offer individuals with more direct information about his/her interpersonal needs range. This goal can be realized through the application of the item response theory (IRT). As the basis of the latest psychometric techniques, the IRT methods can provide analyses of individual latent traits (e.g., interpersonal needs) and item characteristics.

Based on IRT, item and test-information functions can be calculated by integrating the estimated parameters in IRT models to describe graphically and most precisely evaluate the regions of the individual latent trait continuum. Based on the IRT, item and test-information functions assessed on the same latent trait instrument are comparable in different measurements (Fayers, 2004). Therefore, based on the IRT methods, multiple inventories on a single and common metric can be comparable. What is more, the IRT methods can provide suggestions on which item or inventory can provide the most information for different latent traits (Olino et al., 2012).

Until now, no study has compared the psychometric characteristics of different INQ versions based on the IRT in the teenage samples and no study has investigated which version of INQ is more suitable for teenage samples. But the issue of teenagers' suicide cannot be ignored. From the Centers for Disease Control and Prevention, the incidence of suicide attempts peaks in mid-puberty, and the suicide mortality rate steadily increases throughout the teenage period with age. It is the third leading cause of death among young people aged 10–24 (Centers for Disease Control Prevention, 2017). The suicidal characteristics of teenagers are different from those of adults (Parellada et al., 2008). Hence, an effective tool is needed to assess the ranges of interpersonal needs of young people to identify a higher risk of suicidal behaviors. Predicting which individual is likely to commit suicide will help establish strategies for youth suicide prevention and intervention. It is important to explore the potential differences in different versions of INQ to identify the most psychometrically available version to assess the range of interpersonal needs. Moreover, results from many empirical studies found that the relationship between thwarted belongingness and suicidal behaviors was generally weaker in comparison to perceived burdensomeness (Ma et al., 2016; Chu et al., 2017). It is necessary to verify whether this phenomenon exists in teenagers.

In this study, we have investigated and compared the psychometric properties of five versions of the INQ based on an IRT model, to identify the version (or versions), which is more suitable for teenage samples to assess interpersonal needs. Since INQ-15 is a refinement of the INQ-25 (see Van Orden et al., 2012) and how the other versions select items from the original 25-item version is unknown, we hypothesized that the 15-item version would have adequate psychometric characteristics concerning factor structure, internal consistency, and validity. According to Hill et al. (2015), we hypothesized that the 15-item version and 10-item version would show more satisfactory psychometric characteristics compared to the other versions. Based on the IPTS and previous studies, we also hypothesized that in the INQ-12, INQ-18, and INQ-25, perceived burdensomeness, but not thwarted belongingness, would significantly predict capability for suicide and that both PB and TB would significantly predict capability for suicide in the INQ-15 and the INQ-10. Since no research were testing average test information and the differential item functioning caused by gender in the five versions, no specific hypotheses were made. The software R (Version 3.3.21) and the R packages mirt (Version 1.24; Chalmers, 2012) were employed to estimate item parameters. Moreover, we also compared which version could provide greater average test information in a larger range of latent traits. What is more, there was a conversion table provided to obtain the transformed scores of each version. At last, this study is also exempted to test the hypotheses of the IPTS and guide refinement of the IPTS.

## Methods

### Participants

The complete data in the study was available for 905 individuals after deleting the missing response data. Participants were Chinese teenagers from four middle schools in two provinces of China. The mean age of the participants was 15.03 years ranged from 12 to 18 years (*SD* = 1.70). Participants were predominantly male (60.4%), only child (77.8%), and urban (78.6%). Participants were from six grades: Junior One (10.2%), Junior Two (22.9%), Junior Three (10.5%), Senior One (16.6%), Senior Two (16.8%), and Senior Three (23.1%). Both written and verbal consents were acquired from parents of the participants before taking part in the experiment. This study was approved by the Ethics Committee of Jiangxi Normal University and was conducted following the ethical principles of the Declaration of Helsinki.

### Measures

The Interpersonal Needs Questionnaire (INQ; Van Orden, 2009) is a 25-item self-report measure used to assess thwarted belongingness and perceived burdensomeness. Each of the 18-, 15-, 12-, and 10-item versions is a subset of the original 25-item version (see Table 1). Each item is rated on a 7-point Likert scale ranging from 1 (*not at all true for me*) to 7 (*very true for me*). The higher scores represent thwarted belongingness and perceived burdensomeness of the heavier individuals. The coefficients of Cronbach's alpha of five versions ranged from 0.91 to 0.95 in the current study.

**Table 1:** Items included in each version of the INQ.

**Item**	**INQ-25**	**INQ-18**	**INQ-15**	**INQ-12**	**INQ-10**
**Perceived burdensomeness (PB) items**					
1. The people in my life would be better off if I were gone	X	X	X	X	X
2. I think I give back to society	X				
3. The people in my life would be happier without me	X	X	X	X	X
4. I think I have failed the people in my life	X	X		X	
5. I think people in my life would miss me if I went away	X				
6. I think I am a burden on society	X	X	X		
7. I think I am an asset to the people in my life	X				
8. I think my ideas, skills, or energy make a difference	X				
9. I think my death would be a relief to the people in my life	X		X		X
10. I think I contribute to the well-being of the people in my life	X	X		X	
11. I feel like a burden on the people in my life	X	X		X	
12. I think the people in my life wish they could be rid of me	X	X	X	X	X
13. I think I contribute to my community	X				
14. I think I make things worse for the people in my life	X	X	X	X	X
15. I think I matter to the people in my life	X	X			
**Thwarted belongingness (TB) items**					
16. Other people care about me	X	X	X	X	
17. I feel like I belong	X	X	X		X
18. I rarely interact with people who care about me	X	X	X		
19. I am fortunate to have many caring and supportive friends	X	X	X		X
20. I feel disconnected from other people	X	X	X	X	X
21. I often feel like an outsider in social gatherings	X	X	X		X
22. I feel that there are people I can turn to in times of need	X	X	X	X	
23. I feel unwelcome in most social situations	X				
24. I am close to other people	X	X	X	X	X
25. I have at least one satisfying interaction every day	X	X	X	X	

*INQ, Interpersonal Needs Questionnaire; INQ-25, 25-item version of INQ; INQ-18, 18-item version of INQ; INQ-15, 15-item version of INQ; INQ-12, 12-item version of INQ; INQ-10, 10-item version of INQ; X represents an item included in a version*.

The revised UCLA Loneliness Scale (Russell et al., 1980) is a 20-item self-report measure of loneliness. Participants are asked to rate the frequency of satisfaction and dissatisfaction with social relationships. All items are on a 4-point Likert scale ranging from 1 (*never*) to 4 (*always*). The higher scores represent higher levels of loneliness. Russell et al. (1980) reported a high internal consistency for the scale (Cronbach's alpha = 0.94), as well as support for the validity of the scale. In the current study, the scale had a high internal consistency (Cronbach's alpha = 0.91).

The Perceived Social Support Scale (PSSS; Blumenthal et al., 1987) is a 12-item self-report measure of social support. Participants rate their degree of agreement to statements describing people can give them support on a 7-point Likert scale, ranging from “*strongly agree*” to “*strongly disagree*.” The higher scores represent more social support. Blumenthal et al. (1987) reported a good internal consistency (Cronbach's alpha = 0.88). In the current study, the scale had a high internal consistency (Cronbach's alpha = 0.94).

The Acquired Capability for Suicide Scale–Chinese Version (ACSS-CV; Yang et al., 2019) is a 14-item self-report measure of acquired capability for suicide. Participants rate their degree of agreement to statements that describe their fearlessness about lethal self-injury on a 5-point Likert scale, ranging from “*I do not agree at all*” to “*I fully agree*.” The higher scores represent less fearlessness about lethal self-injury. Yang et al. (2019) reported a proper internal consistency (Cronbach's alpha = 0.78). In this study, the scale had a good internal consistency (Cronbach's alpha = 0.80).

### Analysis

#### Construct validity

Confirmatory factor analysis (CFA) was used to examine the fit of the structure of five versions of the INQ, and several global fit indices were used to evaluate the fitness, including the root mean square error of approximation (RMSEA), the standardized root mean square residual (SRMR), the comparative fit index (CFI), and the Tucker–Lewis index (TLI). RMSEA and SRMR values <0.08 (Browne and Cudeck, 1992; Hu and Bentler, 1999), and CFI and TLI values close to 0.95 or greater were considered adequate (Brown, 2006).

#### Internal consistency and validity

Cronbach's alpha coefficients were used to examine the internal consistency of each version scale and its subscales. And regression equations were constructed to test whether the five versions of PB and TB would predict acquired capability for suicide (measured by the ACSS-CV) significantly.

#### Differential item functioning

If respondents from different groups (e.g., gender) with the same ability or proficiency level have different probabilities of choosing the same option for a certain item, then the item is flagged for differential item functioning (DIF; Kim, 2001). In the study, DIF analysis was used to identify systematic bias caused by gender. The IRTPRO program was used to calculate the DIF analysis based on the IRT method. This program performed DIF analysis according to Lord's IRT parameter comparison technique (Lord, 1977) under the framework of IRT.

#### Average test information

Test information is the sum of the information of each item. When the test provides more information to the participants with a certain potential trait value (θ), the standard error of the measurement of these participants will be smaller. In other words, the measurement will be more accurate. Based on the IRT model, we calculated the total test information curve of each subscale separately. The average test information was the total test information divided by the corresponding test length. The equation of item and test information in the graded response model (GRM; Samejima, 1969) are given, respectively, as

(1) $$\begin{array}{r} I_{j}\left(\theta \right)=\overset{mf_{j}}{\underset{t=0}{\sum }}D^{2}a_{j}^{2}\left(P_{jt}^{{}^{*}}-P_{j,t+1}^{*}\right){\left(1-P_{jt}^{*}-P_{j,t+1}^{*}\right)}^{2}, \end{array}$$

and

(2) $$\begin{array}{r} I\left(\theta \right)=\overset{n}{\underset{j=1}{\sum }}I_{j}\left(\theta \right), \end{array}$$

where

(3) $$\begin{array}{r} P_{jt}^{*}=\frac{1}{1+e^{-Da_{j}\left(\theta_{i}-b_{jt}\right)}}. \end{array}$$

Here *a*_*j*_ and *b*_*jt*_ denote the discrimination parameter and the location parameter of the item *j* in GRM, respectively. *b*_*jt*_ is the *t*th location parameter for item *j*, which satisfies *b*_*j*1_ < *b*_*j*2_ < · · · < *b*_*jmf*_*j*__; *mf*_*j*_ represents the maximum score of item *j*. θ_*i*_ refers to the potential trait value of the participant *i*. $P_{jt}^{*}$ denotes the cumulative probability of participants *i* gaining at least a score point *t* on the item *j*. *D* is a constant with a value of 1.7, *I*_*j*_(θ) refers to the information provided by item *j* to participants whose potential trait value is θ, n is the test length. *I*(θ) denotes the total test information.

#### Expected Scores Conversion

Based on the graded response model (GRM), we estimated the item parameters and transferred the potential trait value (θ) to calculate the expected scores of five versions of INQ, and then created a conversion table to implement a comparable process. To calculate the expected scores of subscales, the individual's response probability was calculated based on the GRM. The expected scores for θ_*i*_ can be calculated as

(4) $$\begin{array}{r} Expected\;scores\;\left(\theta_{i}\right)=\overset{n}{\underset{j=1}{\sum }}\overset{mf_{j}}{\underset{t=1}{\sum }}P_{jt}\left(\theta_{i}\right)\times t,\; \end{array}$$

(5) $$\begin{array}{r} P_{jt}\left(\theta_{i}\right)=P_{jt}^{*}-P_{j,t+1}^{*}, \end{array}$$

where *P*_*jt*_(θ_*i*_) is the probability of getting *t* score.

## Result

### Confirmatory Factor Analyses

Consistent with the literature to date, items of each of the five versions were loaded in two dimensions of perceived burdensomeness and thwarted belongingness and were set to load on their hypothesized factor with a correlation between the two factors. Similar to the work of Van Orden et al. (2012), and due to consistently high modification indices across different versions, each subscale had a pair of correlated residuals (items 1 and 3 on the perceived burdensomeness subscales and items 20 and 21 on the thwarted belongingness subscales). Since INQ-12 didn't include both item 20 and item 21, this pair of residuals was not included in the corresponding thwarted belongingness subscale.

Fit indices for the models are presented in Table 2. As can be seen in Table 2, the INQ-25 and the INQ-18 met few criteria for acceptable model fit, which was consistent with Hill et al. (2015), while the INQ-15, the INQ-12, and the INQ-10 met criteria for acceptable fit for all global indices of fit. These results indicated the INQ-15, the INQ-12 and the INQ-10 were more suitable in teenage samples.

**Table 2:** Global fit indices and correlations between subscales.

**Version**	***χ***^**2**^	***df***	**RMSEA**	**CFI**	**TLI**	**SRMR**	**Correlation of PB and TB**
INQ-25	1751.38	272	0.08	0.89	0.88	**0.05**	0.77^**^
INQ-18	718.39	132	**0.07**	0.94	0.93	**0.05**	0.72^**^
INQ-15	347.15	87	**0.06**	**0.96**	**0.96**	**0.04**	0.70^**^
INQ-12	240.63	52	**0.06**	**0.97**	**0.96**	**0.04**	0.66^**^
INQ-10	146.71	32	**0.06**	**0.98**	**0.97**	**0.03**	0.70^**^

*RMSEA, root mean square error of approximation; CFI, comparative fit index; TLI, Tucker–Lewis index; SRMR, standardized root mean square residual; PB, perceived burdensomeness; TB, thwarted belongingness. Bold values indicate acceptable model fit*.

** *p < 0.01; same as below*.

### Internal Consistency and Validity

To examine the internal consistency and criterion validity of each subscale, Cronbach's coefficient alphas and correlation coefficients were generated for each subscale (see Table 3). Both perceived burdensomeness and thwarted belongingness subscales were demonstrated good internal consistency (Cronbach's alphas ranged from 0.81 to 0.93). The internal consistency coefficients of thwarted belongingness subscales were smaller than the corresponding versions of perceived burdensomeness subscales. Both perceived burdensomeness and thwarted belongingness subscales had significant correlation coefficients with the calibration standards of PSSS and UCLA Loneliness Scale. The result verified the views of Van Orden (2009) that interpersonal interactions characterized by low closeness or low frequency could not fully satisfy the sense of belonging, and might lead to feelings of loneliness and perceptions of insufficient social support. The criterion validities between the two subscales of each version were similar.

**Table 3:** Internal consistencies, and correlations between scales.

**Version**	**Internal consistency (Cronbach's alpha)**	**Criterion validity**		
		**UCLA loneliness**	**PSSS**	**ACSS-CV**
INQ-25	0.95	0.75^**^	−0.76^**^	0.16^**^
PB	0.93	0.66^**^	−0.66^**^	0.13^**^
TB	0.89	0.80^**^	−0.80^**^	0.17^**^
INQ-18	0.93	0.77^**^	−0.76^**^	0.15^**^
PB	0.91	0.65^**^	−0.63^**^	0.12^**^
TB	0.87	0.78^**^	−0.79^**^	0.17^**^
INQ-15	0.92	0.78^**^	−0.78^**^	0.18^**^
PB	0.90	0.64^**^	−0.62^**^	0.16^**^
TB	0.87	0.78^**^	−0.79^**^	0.17^**^
INQ-12	0.91	0.76^**^	−0.75^**^	0.15^**^
PB	0.90	0.66^**^	−0.64^**^	0.13^**^
TB	0.81	0.75^**^	−0.76^**^	0.15^**^
INQ-10	0.91	0.76^**^	−0.74^**^	0.19^**^
PB	0.90	0.65^**^	−0.62^**^	0.17^**^
TB	0.81	0.76^**^	−0.75^**^	0.18^**^

*PSSS, Perceived Social Support Scale; ACSS-CV, Acquired Capability for Suicide Scale–Chinese Version*.

** *p < 0.01*.

To examine concurrent predictive validity, the regression equations were conducted. Both perceived burdensomeness and thwarted belongingness of each version were significant predictors of acquired capability for suicide (see Table 4).

**Table 4:** Regression models of perceived burdensomeness and thwarted belongingness predicting acquired capability for suicide.

**Model**		***R^**2**^***	***F***	***t***	***p***
INQ-25	PB	0.018	16.284	4.035	0.000
	TB	0.029	26.942	5.191	0.000
INQ-18	PB	0.014	13.132	3.624	0.000
	TB	0.029	26.833	5.180	0.000
INQ-15	PB	0.026	23.746	4.873	0.000
	TB	0.029	26.833	5.180	0.000
INQ-12	PB	0.017	15.624	3.953	0.000
	TB	0.023	20.848	4.566	0.000
INQ-10	PB	0.029	26.650	5.162	0.000
	TB	0.032	30.176	5.493	0.000

### Differential Item Functioning

To examine the differential item functioning of 25 items, the ‘gender’ variable divided into males and females was used to analyze. According to parameter comparison, the first item with the significance level of 0.01 and the 18th item with the significance level of 0.001 existed DIF (see Table 5). All versions contained the first item. Therefore, the versions without the 18th item were suggested such as the 10-item version and the 12-item version.

**Table 5:** DIF statistics for graded response model.

**Item**	**Total *X*^**2**^**	***d.f*.**	***p***	$X_{\text{a}}^{2}$	***d.f*.**	***p***	$X_{\text{c}|\text{a}}^{2}$	***d.f*.**	***p***
1	21.2	7	0.0035	2.6	1	0.1091	18.6	6	0.0049^**^
2	9.5	7	0.2208	0.4	1	0.5193	9	6	0.1707
3	12	7	0.0993	3.4	1	0.0666	8.7	6	0.1927
4	5.1	7	0.6538	0	1	0.871	5	6	0.5409
5	19.8	7	0.006	3.3	1	0.0681	16.5	6	0.0114
6	7.5	7	0.3841	1.8	1	0.1779	5.6	6	0.4663
7	11.4	7	0.1218	1	1	0.3107	10.4	6	0.1096
8	5.8	7	0.5611	0.1	1	0.7006	5.7	6	0.4613
9	4.5	7	0.7207	0.1	1	0.7507	4.4	6	0.623
10	8.6	7	0.2839	4.1	1	0.0441	4.6	6	0.6029
11	5.2	7	0.6381	0.4	1	0.5063	4.7	6	0.578
12	3.9	7	0.7866	0.8	1	0.3662	3.1	6	0.7934
13	13.6	7	0.0582	4	1	0.0455	9.6	6	0.1411
14	12.3	7	0.0912	3.8	1	0.0524	8.5	6	0.2014
15	4	7	0.7857	0	1	0.933	3.9	6	0.6847
16	10.3	7	0.1695	0.1	1	0.8068	10.3	6	0.1129
17	6.4	7	0.4954	1.6	1	0.2029	4.8	6	0.5746
18	26.9	7	0.0004	0.1	1	0.8133	26.8	6	0.0002^***^
19	16	7	0.0252	0.2	1	0.6267	15.8	6	0.0151
20	14	7	0.0516	2.2	1	0.1416	11.8	6	0.0665
21	20.1	7	0.0053	5.3	1	0.0214	14.8	6	0.0217
22	13.3	7	0.0651	5.2	1	0.0227	8.1	6	0.2298
23	15.9	7	0.0263	7.7	1	0.0056	8.2	6	0.2239
24	14.3	7	0.0463	0.6	1	0.4339	13.7	6	0.0335
25	10.6	7	0.1591	0.1	1	0.7263	10.4	6	0.1075

*Total X^2^, total variance; X^2a^, within-group variance; ${\text{X}}_{\text{c}|\text{a}}^{2}$, between-group variance*.

** *p < 0.01*.

*** *p < 0.001*.

### Average Test Information Curve

**Figure 1:**
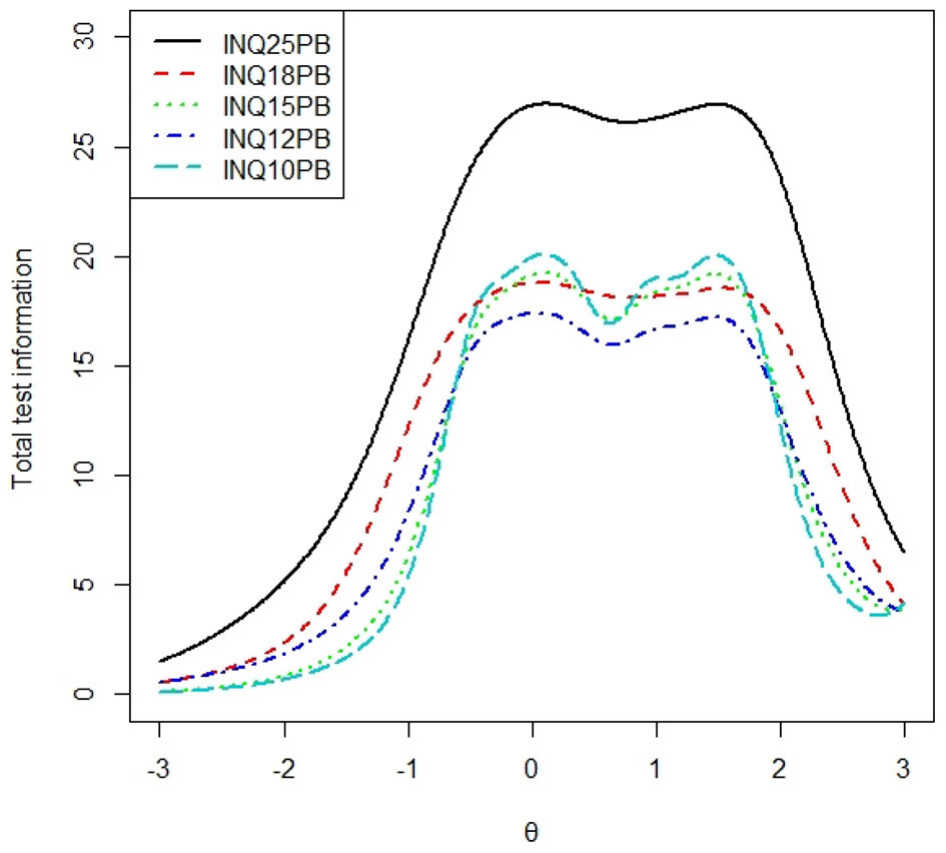
Total test information curves of perceived burdensomeness of each version. INQ25PB, perceived burdensomeness subscale of INQ-25; INQ18PB, perceived burdensomeness subscale of INQ-18; INQ15PB, perceived burdensomeness subscale of INQ-15; INQ12PB, perceived burdensomeness subscale of INQ-12; INQ10PB, perceived burdensomeness subscale of INQ-10, same as below.

**Figure 2:**
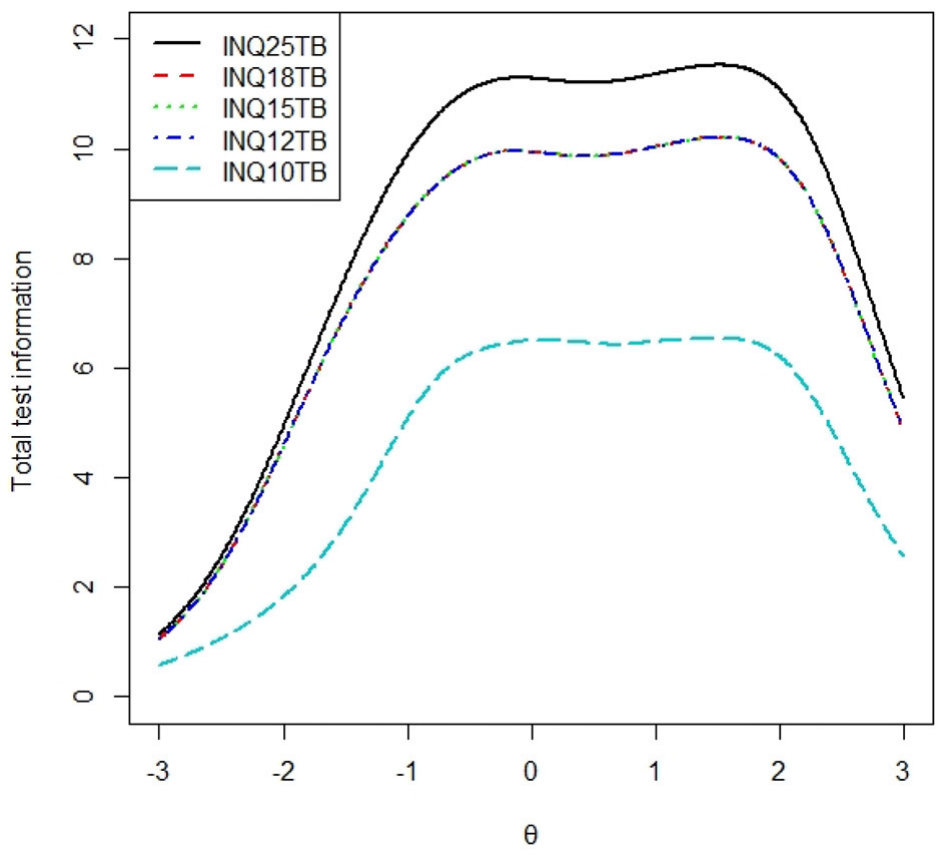
Total test information curves of thwarted belongingness of each version. INQ25TB=thwarted belongingness subscale of INQ-25; INQ18TB=thwarted belongingness subscale of INQ-18; INQ15TB=thwarted belongingness subscale of INQ-15; INQ12TB=thwarted belongingness subscale of INQ-12; INQ10TB=thwarted belongingness subscale of INQ-10, same as below.

**Figure 3:**
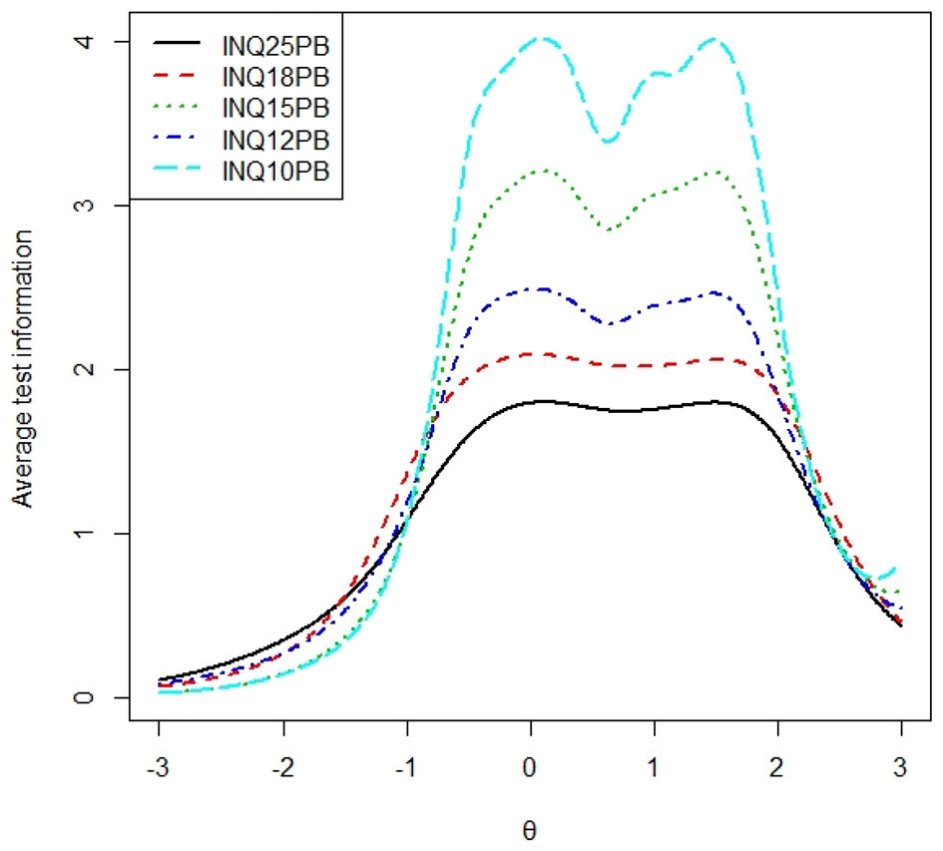
Average test information curves of perceived burdensomeness of each version.

**Figure 4:**
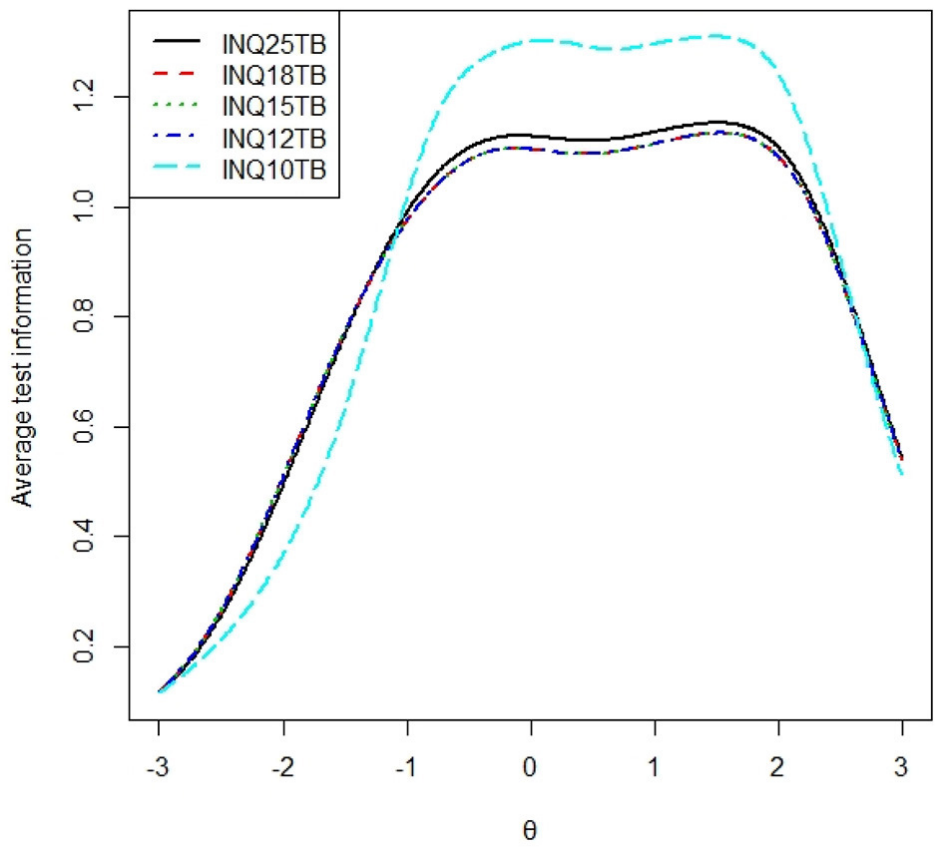
Average test information curves of thwarted belongingness of each version.

To examine the average test information of each subscale, we calculated the total test information curve of each subscale presented in Figures 1, 2, and the average test information curves were presented in Figures 3, 4, which indicated the item information contained at each node along the θ scale. Measurement providing more information had higher reliability and more measurement precision. In Figure 3, the INQ-10 provided the most average test information at the range approximately from −0.8 to 2 standard deviations of perceived burdensomeness, among the five versions. At high ranges of θ value, the five scales provided a similar amount of information, while at other ranges of θ value, the INQ-10 provided the least in the five scales. On the whole, the five scales could provide proper average test information. The results suggested that the INQ-10 could provide more measurement precision for varying degrees of perceived burdensomeness, and this suggested that the INQ-10 might be more useful in teenage samples for measuring perceived burdensomeness in clinical trials and measuring perceived burdensomeness as an index of treatment response. In Figure 4, above −1 standard deviations of thwarted belongingness, the INQ-10 provided the most average test information, while at other ranges of θ value, the INQ-10 was the least in the five scales, but close to the other versions. Similar to the result of perceived burdensomeness subscales, the thwarted belongingness subscale of the INQ-10 could provide higher reliability and more measurement precision for varying degrees of thwarted belongingness. Overall, both the perceived burdensomeness subscale and the thwarted belongingness subscale of the INQ-10 had the highest reliability and most measurement precision. Hence, in the five versions of INQ, choosing the INQ-10 may be better in teenage samples at varying degrees of interpersonal needs.

### Expected Scores

The expected scores of the five versions were calculated by transferring θ values based on GRM and presented in Table 6. The scores conversion means that the scores of the five versions measuring the same psychological trait can be compared with each other. The conversion table of the five scale scores provides help shifting one scale into another one and is useful for future study and application when different version scores need to be switched.

**Table 6:** Conversion table of five versions of INQ based on expected scores.

**θ Scale**	**Perceived burdensomeness**						**Thwarted belongingness**				
	**INQ25**	**INQ18**	**INQ15**	**INQ12**	**INQ10**		**INQ25**	**INQ18**	**INQ15**	**INQ12**	**INQ10**
−3	15.23	9.04	6.00	7.04	5.00		10.06	9.05	9.05	5.04	5.02
−2.9	15.29	9.05	6.00	7.05	5.00		10.08	9.07	9.07	5.05	5.03
−2.8	15.37	9.06	6.00	7.06	5.00		10.10	9.10	9.10	5.07	5.04
−2.7	15.47	9.08	6.00	7.08	5.00		10.14	9.13	9.13	5.10	5.05
−2.6	15.59	9.11	6.00	7.11	5.00		10.19	9.18	9.18	5.14	5.07
−2.5	15.75	9.14	6.00	7.14	5.00		10.25	9.24	9.24	5.19	5.10
−2.4	15.94	9.19	6.00	7.19	5.00		10.34	9.33	9.33	5.25	5.14
−2.3	16.17	9.25	6.00	7.24	5.00		10.46	9.44	9.44	5.34	5.18
−2.2	16.45	9.32	6.01	7.32	5.00		10.61	9.58	9.58	5.46	5.25
−2.1	16.79	9.42	6.01	7.41	5.00		10.81	9.77	9.77	5.62	5.33
−2	17.19	9.53	6.01	7.52	5.01		11.06	10.01	10.01	5.81	5.44
−1.9	17.66	9.68	6.02	7.66	5.01		11.37	10.31	10.31	6.05	5.58
−1.8	18.20	9.85	6.03	7.83	5.02		11.76	10.67	10.67	6.35	5.75
−1.7	18.81	10.06	6.04	8.02	5.03		12.22	11.09	11.09	6.69	5.94
−1.6	19.50	10.31	6.06	8.24	5.04		12.75	11.58	11.58	7.08	6.17
−1.5	20.27	10.59	6.09	8.50	5.07		13.36	12.14	12.14	7.51	6.43
−1.4	21.11	10.92	6.14	8.78	5.11		14.06	12.77	12.77	7.99	6.72
−1.3	22.03	11.30	6.22	9.11	5.17		14.83	13.46	13.46	8.51	7.04
−1.2	23.04	11.76	6.33	9.48	5.26		15.68	14.22	14.22	9.06	7.41
−1.1	24.16	12.31	6.49	9.91	5.39		16.62	15.05	15.05	9.64	7.82
−1	25.40	12.96	6.71	10.41	5.57		17.62	15.94	15.94	10.24	8.28
−0.9	26.78	13.75	7.02	11.01	5.83		18.70	16.89	16.89	10.87	8.79
−0.8	28.32	14.68	7.43	11.73	6.17		19.83	17.90	17.90	11.50	9.34
−0.7	30.01	15.77	7.96	12.58	6.61		21.02	18.95	18.95	12.15	9.94
−0.6	31.87	17.01	8.62	13.55	7.16		22.26	20.06	20.06	12.79	10.57
−0.5	33.87	18.39	9.43	14.63	7.84		23.54	21.19	21.19	13.45	11.23
−0.4	36.00	19.87	10.36	15.79	8.62		24.85	22.36	22.36	14.11	11.92
−0.3	38.24	21.43	11.40	17.01	9.50		26.19	23.56	23.56	14.77	12.62
−0.2	40.58	23.06	12.55	18.28	10.48		27.54	24.77	24.77	15.42	13.35
−0.1	42.99	24.74	13.77	19.58	11.52		28.90	25.97	25.97	16.06	14.08
0	45.45	26.45	15.05	20.91	12.61		30.24	27.17	27.17	16.68	14.82
−0.1	47.91	28.16	16.35	22.24	13.72		31.57	28.36	28.36	17.28	15.56
−0.2	50.35	29.85	17.66	23.55	14.84		32.88	29.52	29.52	17.85	16.29
−0.3	52.74	31.50	18.94	24.82	15.93		34.18	30.67	30.67	18.41	17.01
−0.4	55.06	33.07	20.18	26.03	16.98		35.46	31.81	31.81	18.95	17.72
−0.5	57.28	34.56	21.34	27.17	17.96		36.73	32.94	32.94	19.50	18.42
−0.6	59.40	35.96	22.41	28.24	18.87		38.00	34.07	34.07	20.05	19.13
−0.7	61.47	37.31	23.42	29.27	19.72		39.27	35.21	35.21	20.62	19.83
−0.8	63.53	38.67	24.40	30.32	20.55		40.57	36.36	36.36	21.21	20.54
−0.9	65.63	40.05	25.38	31.40	21.39		41.89	37.54	37.54	21.83	21.26
1	67.79	41.47	26.41	32.52	22.28		43.24	38.75	38.75	22.47	21.99
1.1	70.01	42.90	27.49	33.67	23.21		44.63	40.00	40.00	23.13	22.74
1.2	72.31	44.36	28.61	34.84	24.19		46.05	41.27	41.27	23.82	23.50
1.3	74.70	45.87	29.78	36.04	25.20		47.50	42.58	42.58	24.53	24.27
1.4	77.19	47.45	31.00	37.31	26.26		48.99	43.92	43.92	25.26	25.06
1.5	79.78	49.10	32.28	38.63	27.37		50.51	45.29	45.29	26.01	25.87
1.6	82.43	50.78	33.59	39.97	28.51		52.05	46.68	46.68	26.78	26.69
1.7	85.09	52.46	34.91	41.31	29.66		53.62	48.10	48.10	27.56	27.52
1.8	87.71	54.10	36.19	42.60	30.75		55.19	49.53	49.53	28.35	28.35
1.9	90.24	55.67	37.38	43.82	31.75		56.76	50.95	50.95	29.13	29.18
2	92.59	57.11	38.43	44.92	32.62		58.31	52.36	52.36	29.91	29.99
2.1	94.72	58.39	39.31	45.87	33.31		59.81	53.72	53.72	30.66	30.76
2.2	96.57	59.47	40.01	46.65	33.84		61.24	55.02	55.02	31.37	31.49
2.3	98.15	60.34	40.56	47.26	34.22		62.56	56.21	56.21	32.02	32.15
2.4	99.48	61.03	40.97	47.73	34.49		63.74	57.27	57.27	32.60	32.72
2.5	100.58	61.55	41.27	48.07	34.67		64.78	58.21	58.21	33.10	33.21
2.6	101.48	61.94	41.49	48.32	34.79		65.67	59.01	59.01	33.52	33.62
2.7	102.22	62.24	41.65	48.51	34.87		66.42	59.68	59.68	33.86	33.94
2.8	102.81	62.45	41.76	48.64	34.92		67.04	60.24	60.24	34.13	34.20
2.9	103.29	62.60	41.83	48.74	34.95		67.55	60.70	60.70	34.35	34.40
3	103.67	62.71	41.89	48.81	34.97		67.97	61.08	61.08	34.51	34.55

## Discussion

To date, the present study provides the first comparison of five versions of INQ simultaneously within teenage samples. Construct validity, internal consistency, validity, and average test information were compared for the five versions to (a) identify the version with the optimal overall psychometric characteristics in teenage samples to encourage future use, and (b) test the hypotheses of the IPTS and guide refinement of the IPTS.

Concerning validity, the INQ-15, INQ-12, and INQ-10 demonstrated adequate fit for a two-factor model for all global indices of fit, while the other longer versions did not. Thus, the INQ-15, INQ-12, and INQ-10 most consistently demonstrated construct validity, providing evidence in support of their continued use in teenage samples. The results of internal consistency and criterion validity showed that five versions of the INQ and their subscales all had good internal consistency. But the internal consistency coefficients of thwarted belongingness subscales were smaller than perceived burdensomeness subscales, which was similar to most previous researches (e.g., Bryan et al., 2010; Hill and Pettit, 2012; Monteith et al., 2013; Teo et al., 2018). The correlation coefficients of perceived burdensomeness and thwarted belongingness subscales were closed to each other. As for concurrent predictive validity, both perceived burdensomeness and thwarted belongingness of each version were significant predictors of acquired capability for suicide. The internal consistency, correlation coefficient, and concurrent validity of different versions did not provide any basis for recommending a specific version of the INQ. According to the DIF analysis, the first item with the significance level of 0.01 and 18th with the significance level of 0.001 item had DIF. All versions contained the first item. Therefore, the versions without the 18th item were suggested such as the 10-item version and the 12-item version. As for average test information, the INQ-10 had clear advantages both in the perceived burdensomeness subscale and in thwarted belongingness subscale. In perceived burdensomeness subscales, the version that included more items provided less average test information. In thwarted belongingness subscales, the thwarted belongingness subscale of INQ-10 evidently provided the most average test information, and the thwarted belongingness subscale of INQ-25 provided the second most information. The average test information curves of thwarted belongingness subscales of the other three versions almost coincided. The thwarted belongingness subscales of INQ-18 and INQ-15 contained the same items, and the result demonstrated that 5-item of thwarted belongingness subscale of the INQ-12 provided the same average test information with 9-item of thwarted belongingness subscales of the INQ-15 and the INQ-18. Concerning the expected scores, the conversion of the five scale scores (see Table 6) enables the conversion of one scale shifting into another one. The conversion table can provide help for future studies and applications when one of the five scale scores needs to be transformed into another.

Overall, the result of average test information suggested the INQ-10 provided higher reliability and more measurement precision, and the 10-item version with proper reliability and validity was demonstrated as an adequate fit for a two-factor model. Hence, the 10-item version of INQ is the most suitable version for future use in teenage samples. In addition, the results above showed that perceived burdensomeness performed better in multiple indicators in comparison to thwarted belongingness. If different results are a consequence of measurement, it is necessary to take into consideration both theoretical and operational definitions of the thwarted belongingness and perceived burdensomeness. It is also possible that the thwarted belongingness subscale of the INQ is not adequately measuring the thwarted belongingness and developing a new self-report scale for thwarted belongingness is needed.

The results of this study should be viewed within the context of its limitations. First, the present study uses data from a teenage sample in China, which limits the generalizability of the results. According to the study, we cannot make a decision on which version of the INQ is best for the elderly and clinical samples. Furthermore, the INQ was demonstrated that it could predict suicidal ideation of the individual in previous studies (Joiner, 2005; Van Orden et al., 2010). But this study demonstrated that the INQ could predict the acquired capability for suicide and it was cross-sectional and did not examine the difference of predictive validity about suicidal ideation among the five versions, which can be done in the future. In addition, the five versions of INQ were derived from the response to INQ-25. Thus, current data did not take into account the possible influence of question order effects (e.g., consecutive questions might be answered more similarly than non-continuous questions). Furthermore, the internal consistency coefficients of thwarted belongingness subscales were smaller than the perceived burdensomeness subscales in this study. And findings in previous studies for the relationship between thwarted belongingness and suicidal ideation were weaker in comparison to perceived burdensomeness (Ma et al., 2016; Chu et al., 2017). In the future study, a new self-report scale for thwarted belongingness (TB) can be developed to expand the availability of valid measurement approaches for interpersonal risk.

## Data Availability Statement

The raw data supporting the conclusions of this article will be made available by the authors, without undue reservation.

## Ethics Statement

The studies involving human participants were reviewed and approved by Ethics Committee of School of Psychology, Jiangxi Normal University. Written informed consent to participate in this study was provided by the participants' legal guardian/next of kin.

## Author Contributions

DT, XY, and YC selected the topic and made some modifications of the paper. JQ collected the data and wrote the manuscript. All authors contributed to the article and approved the submitted version.

## Conflict of Interest

The authors declare that the research was conducted in the absence of any commercial or financial relationships that could be construed as a potential conflict of interest.
