# Supplementary material for: Comparison of Psychometric Characteristics for Five Versions of the Interpersonal Needs Questionnaire in Teenagers Sample
Source: Front Psychol. 2021 May 28;12:676361. doi: 10.3389/fpsyg.2021.676361 (PMC8193059; doi:10.3389/fpsyg.2021.676361)
Supplement: Supplementary file 1 [file Image_1.pdf]

Appendix

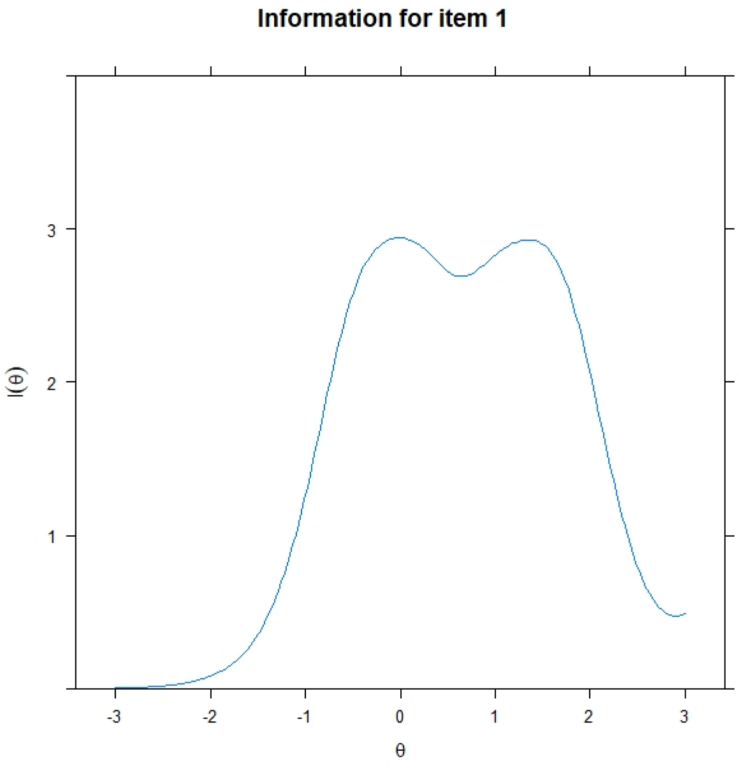

Figure 1

Item Information Curves of Perceived Burdensomeness, Item 1

Note. Item 1 of perceived burdensomeness=Item 1 of the INQ-25.

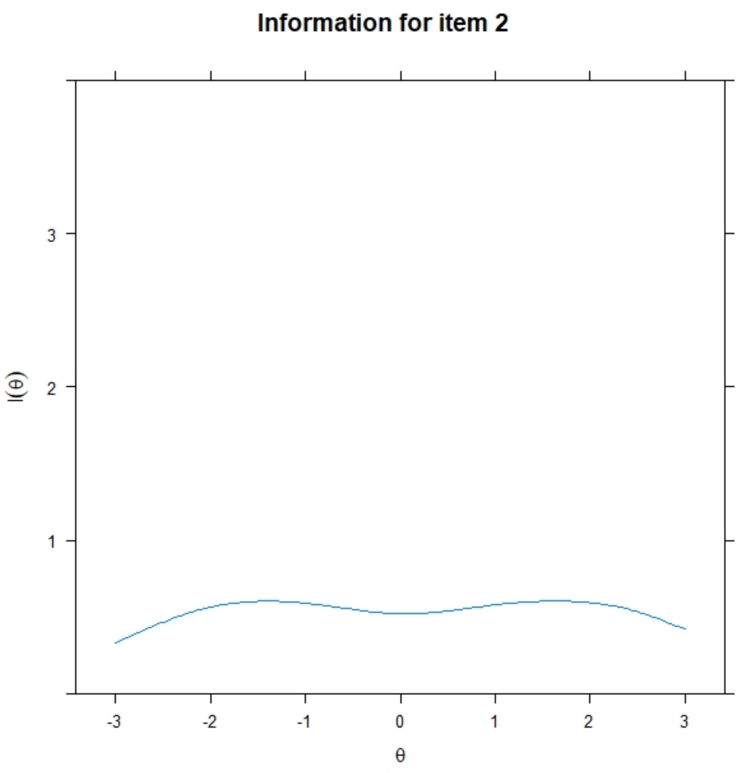

Figure 2

Item Information Curves of Perceived Burdensomeness, Item 2

Note. Item 2 of perceived burdensomeness=Item 2 of the INQ-25.

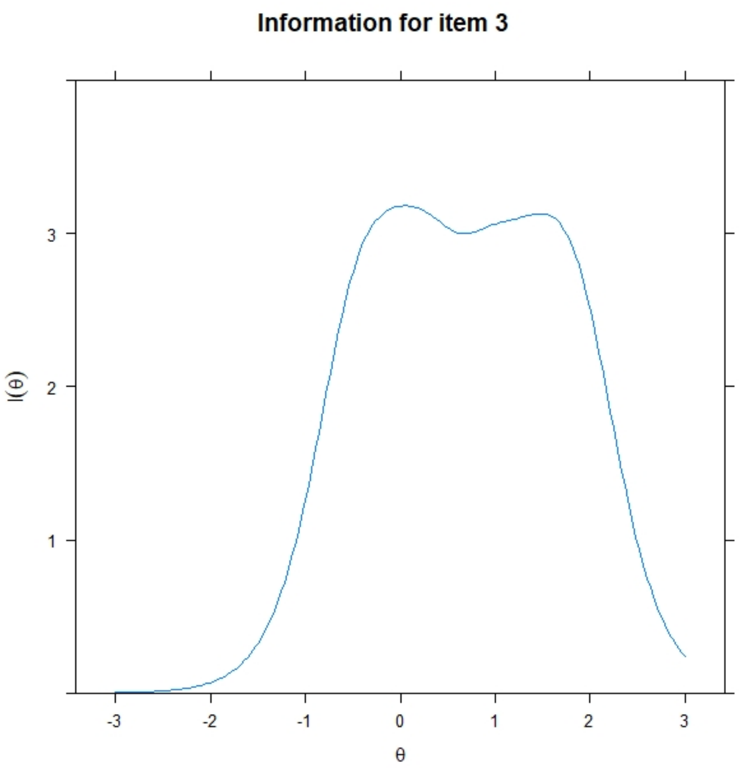

Figure 3

Item Information Curves of Perceived Burdensomeness, Item 3

Note. Item 3 of perceived burdensomeness=Item 3 of the INQ-25.

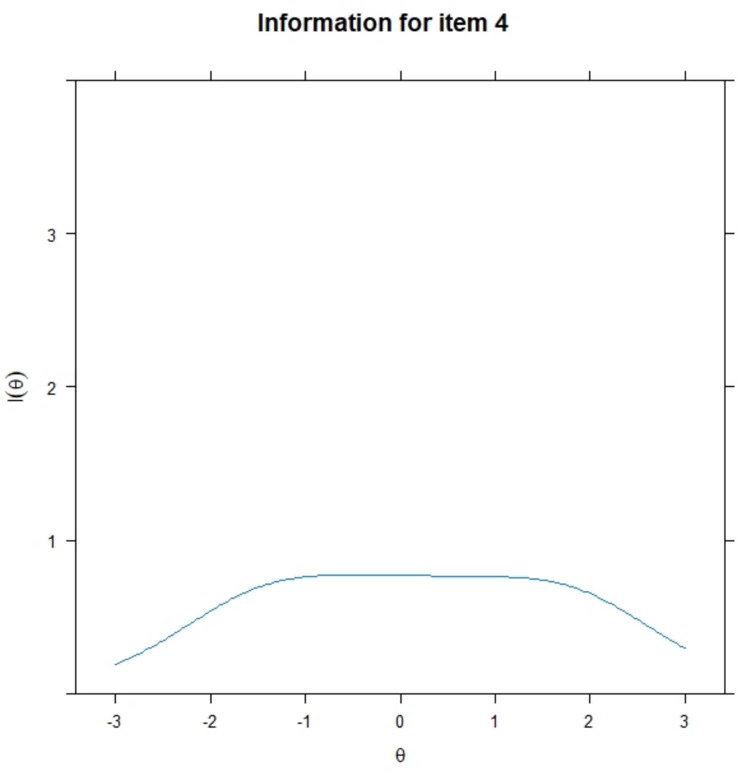

Figure 4

Item Information Curves of Perceived Burdensomeness, Item 4

Note. Item 4 of perceived burdensomeness=Item 4 of the INQ-25.

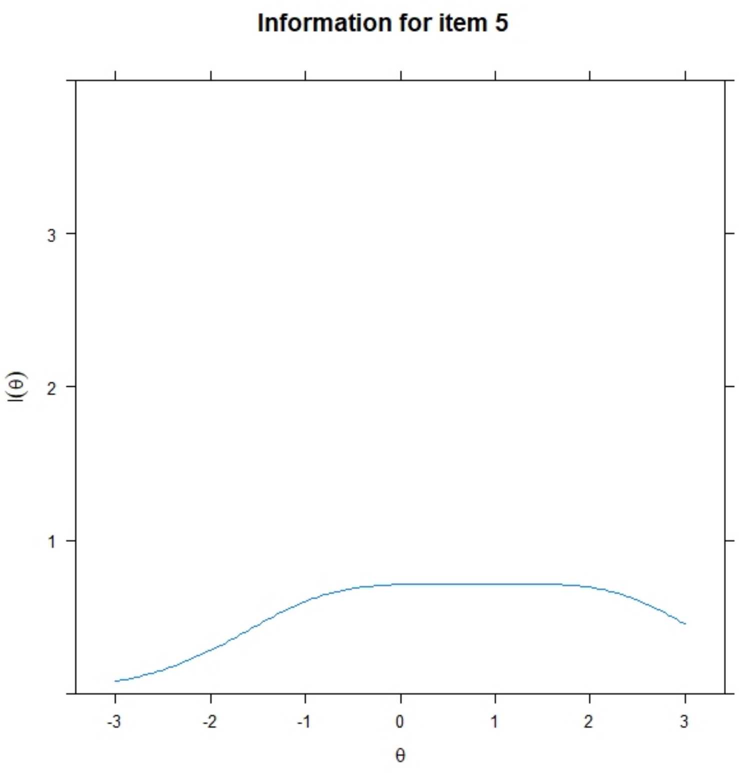

Figure 5

Item Information Curves of Perceived Burdensomeness, Item 5

Note. Item 5 of perceived burdensomeness=Item 5 of the INQ-25.

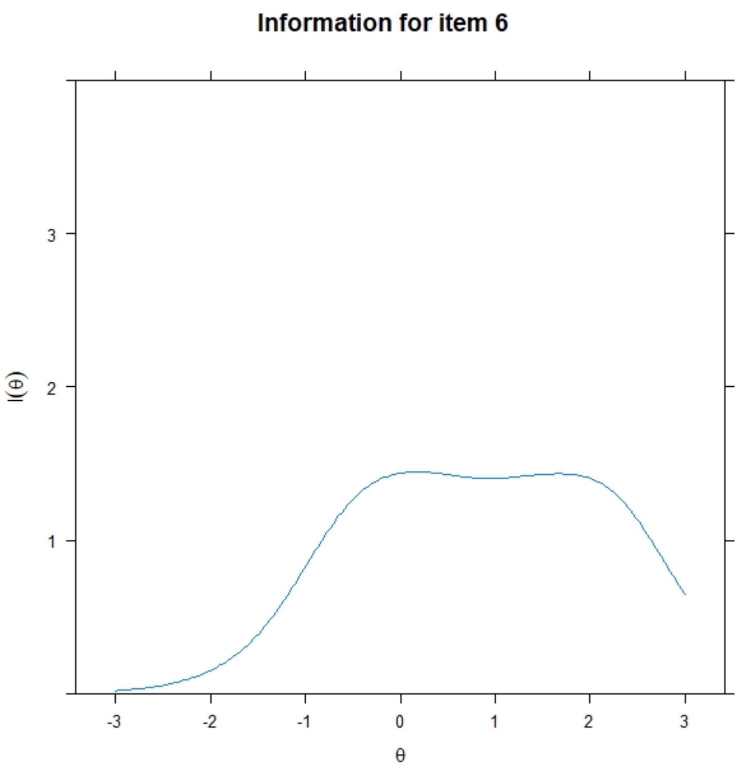

Figure 6

Item Information Curves of Perceived Burdensomeness, Item 6

Note. Item 6 of perceived burdensomeness=Item 6 of the INQ-25.

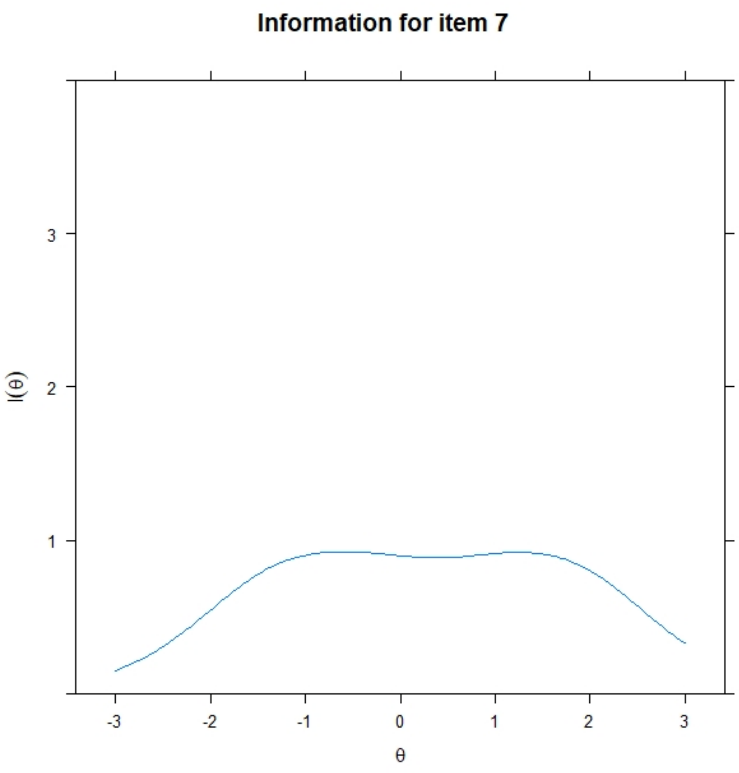

Figure 7

Item Information Curves of Perceived Burdensomeness, Item 7

Note. Item 7 of perceived burdensomeness=Item 7 of the INQ-25.

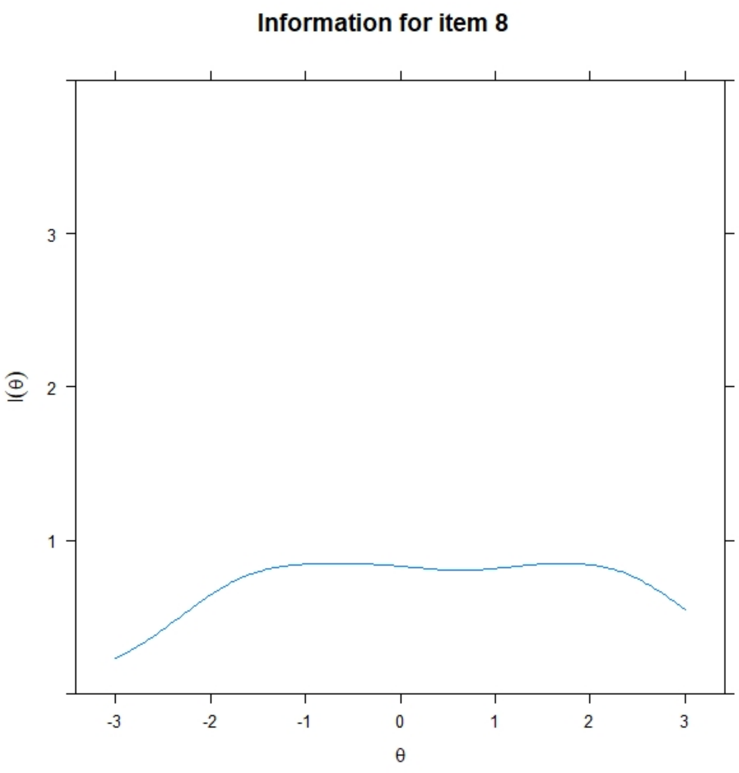

Figure 8

Item Information Curves of Perceived Burdensomeness, Item 8

Note. Item 8 of perceived burdensomeness=Item 8 of the INQ-25.

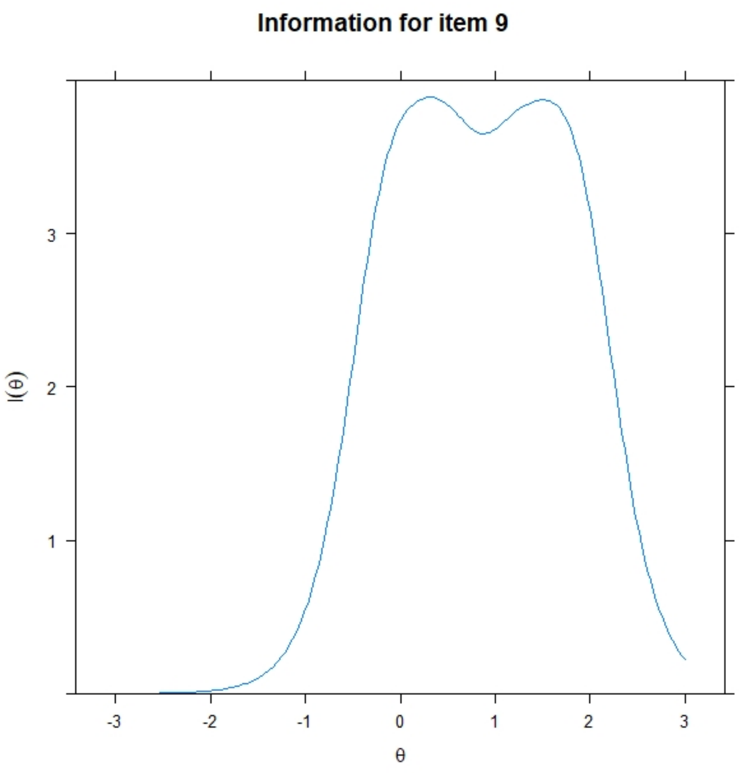

Figure 9

Item Information Curves of Perceived Burdensomeness, Item 9

Note. Item 9 of perceived burdensomeness=Item 9 of the INQ-25.

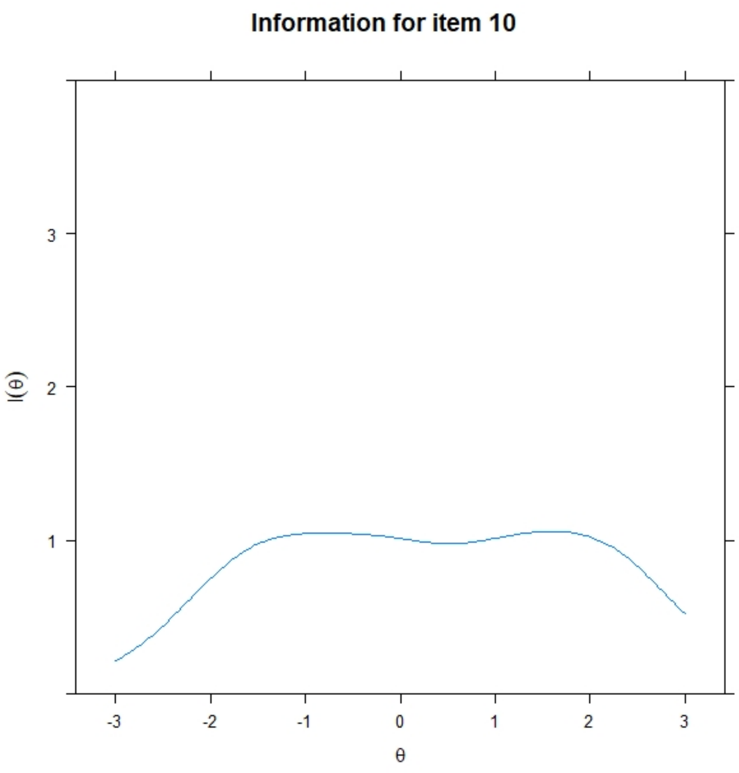

Figure 10

Item Information Curves of Perceived Burdensomeness, 1tem 10

Note. Item 10 of perceived burdensomeness=Item 10 of the INQ-25.

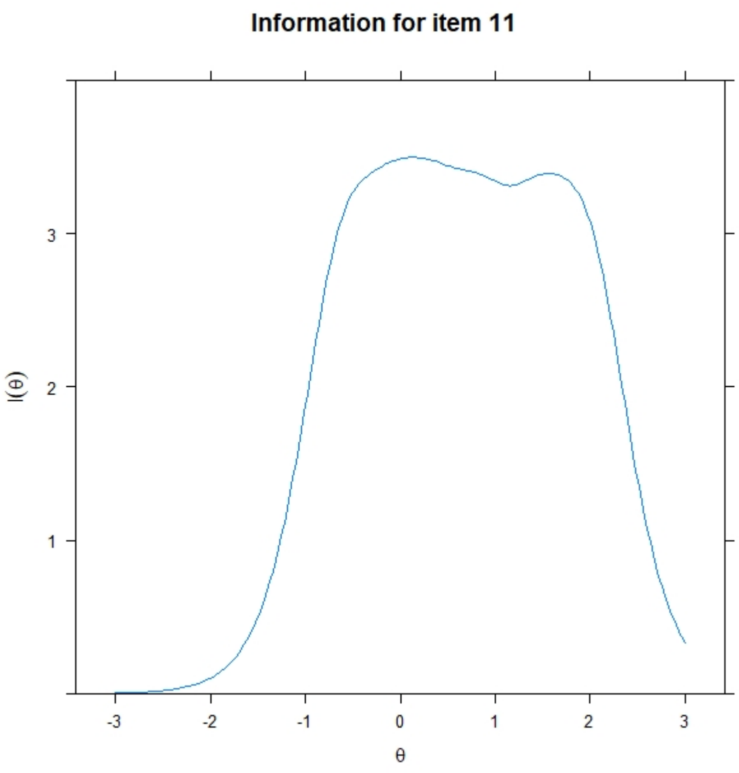

Figure 11

Item Information Curves of Perceived Burdensomeness, Item 11

Note. Item 11 of perceived burdensomeness=Item 11 of the INQ-25.

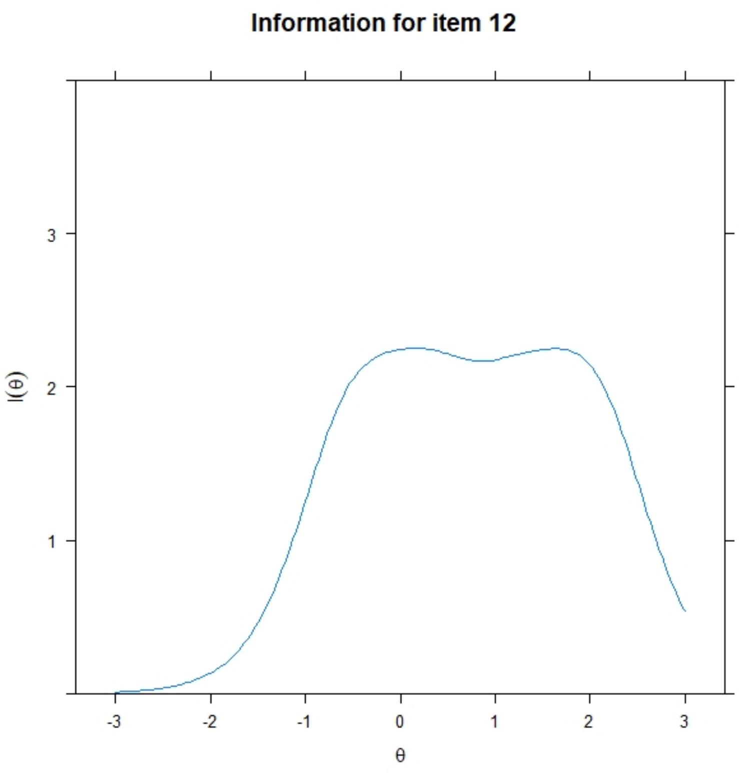

Figure 12

Item Information Curves of Perceived Burdensomeness, Item 12

Note. Item 12 of perceived burdensomeness=Item 12 of the INQ-25.

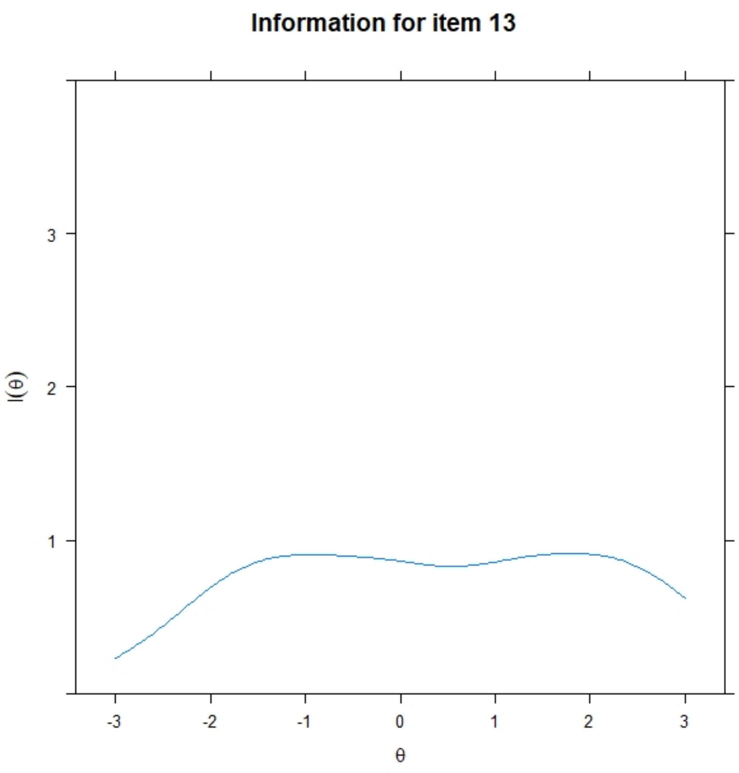

Figure 13

Item Information Curves of Perceived Burdensomeness, Item 13

Note. Item 13 of perceived burdensomeness=Item 13 of the INQ-25.

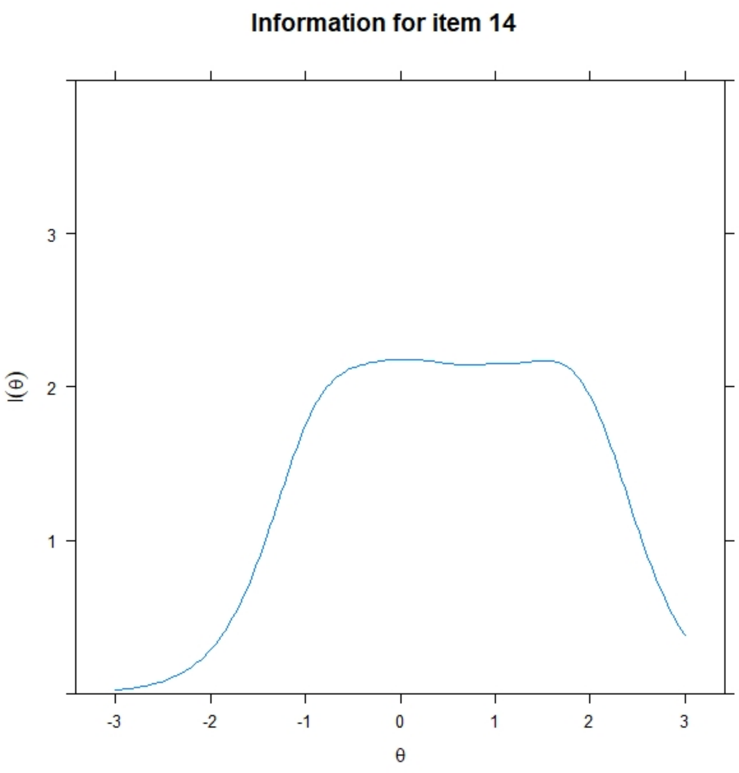

Figure 14

Item Information Curves of Perceived Burdensomeness, Item 14

Note. Item 14 of perceived burdensomeness=Item 14 of the INQ-25.

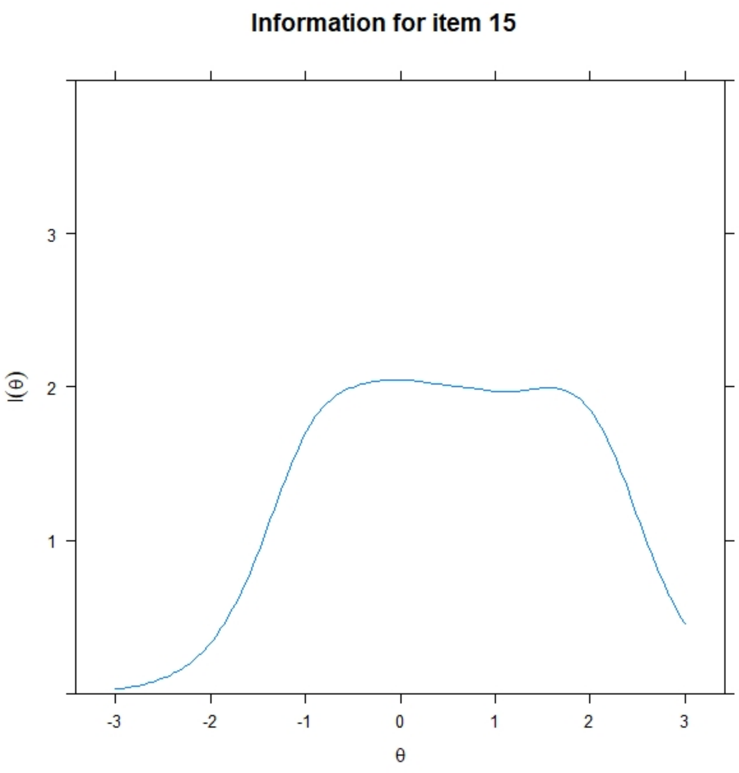

Figure 15

Item Information Curves of Perceived Burdensomeness, Item 15

Note. Item 15 of perceived burdensomeness=Item 15 of the INQ-25.

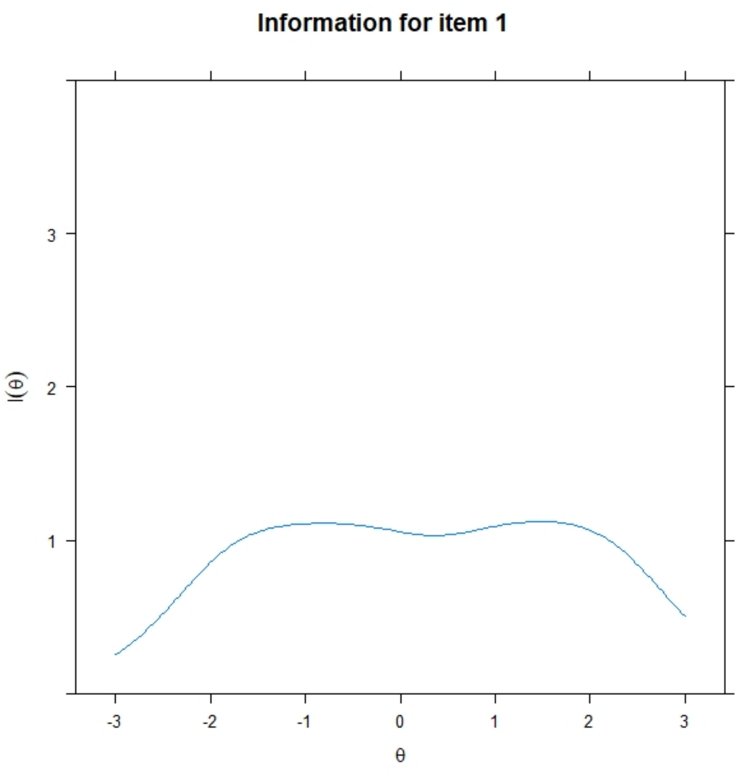

Figure 16

Item Information Curves of Thwarted Belongingness, Item 1

Note. Item 1of thwarted belongingness=Item 16 of the INQ-25.

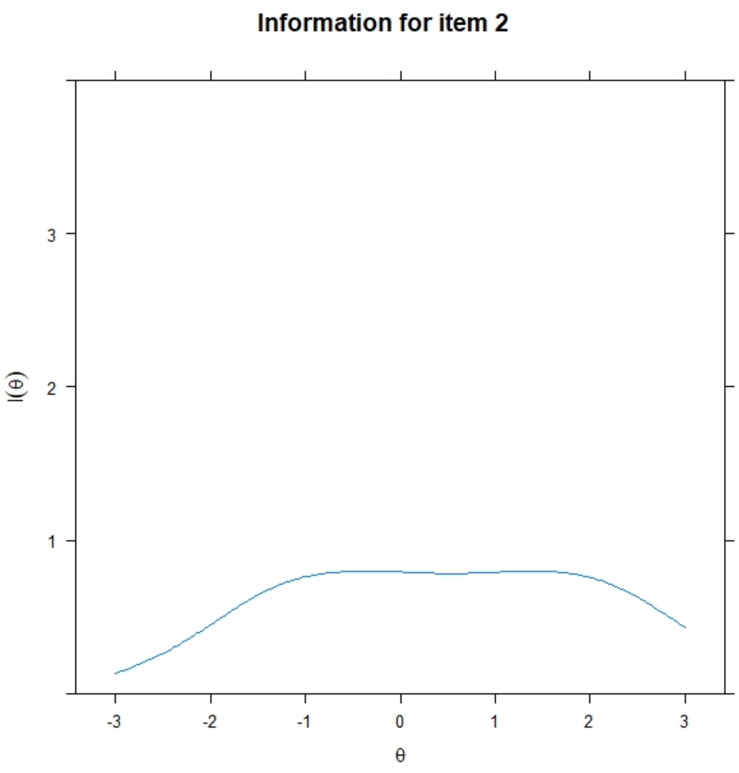

Figure 17

Item Information Curves of Thwarted Belongingness, Item 2

Note. Item 2 of thwarted belongingness=Item 17 of the INQ-25.

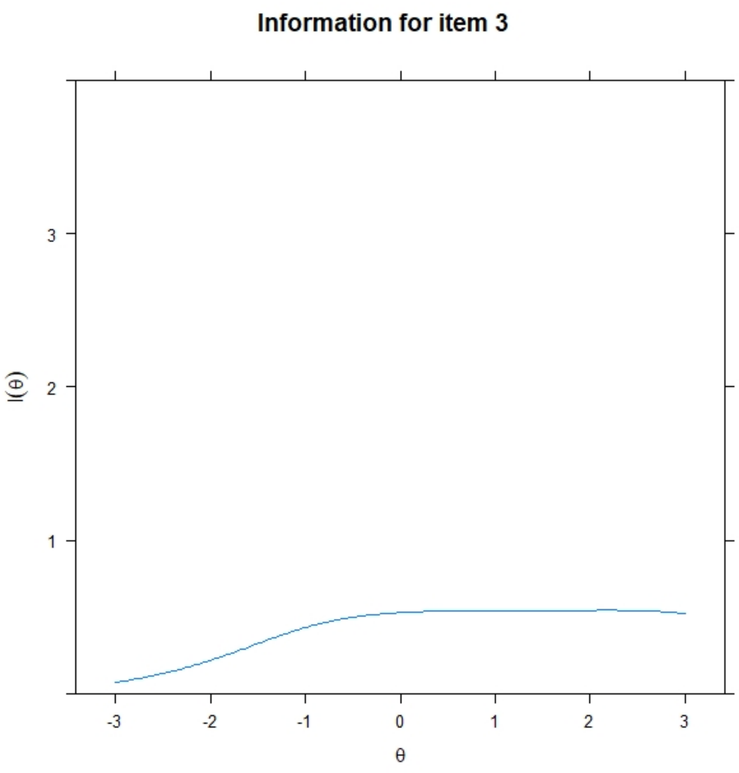

Figure 18

Item Information Curves of Thwarted Belongingness, Item 3

Note. Item 3 of thwarted belongingness=Item 18 of the INQ-25.

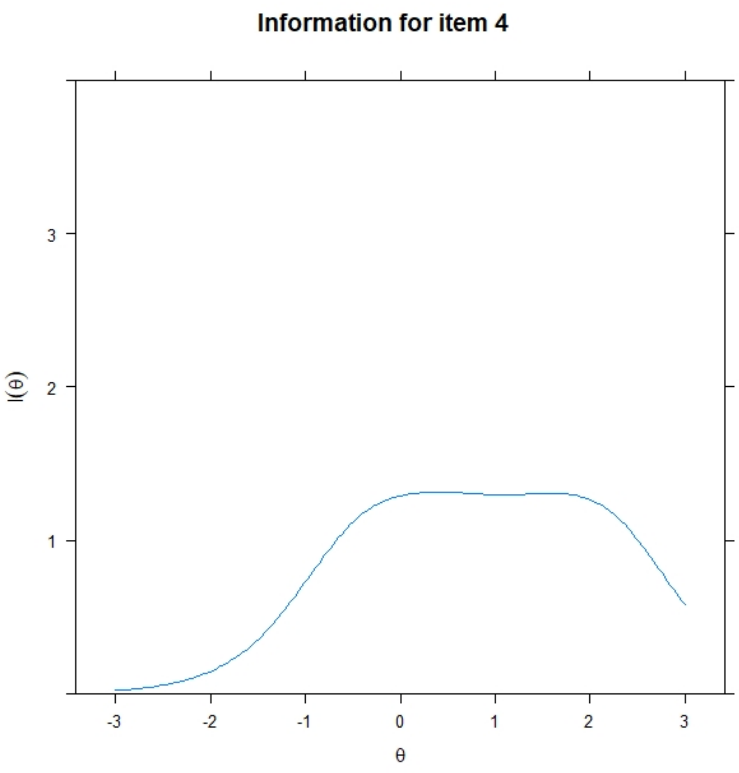

Figure 19

Item Information Curves of Thwarted Belongingness, Item 4

Note. Item 4 of thwarted belongingness=Item 19 of the INQ-25.

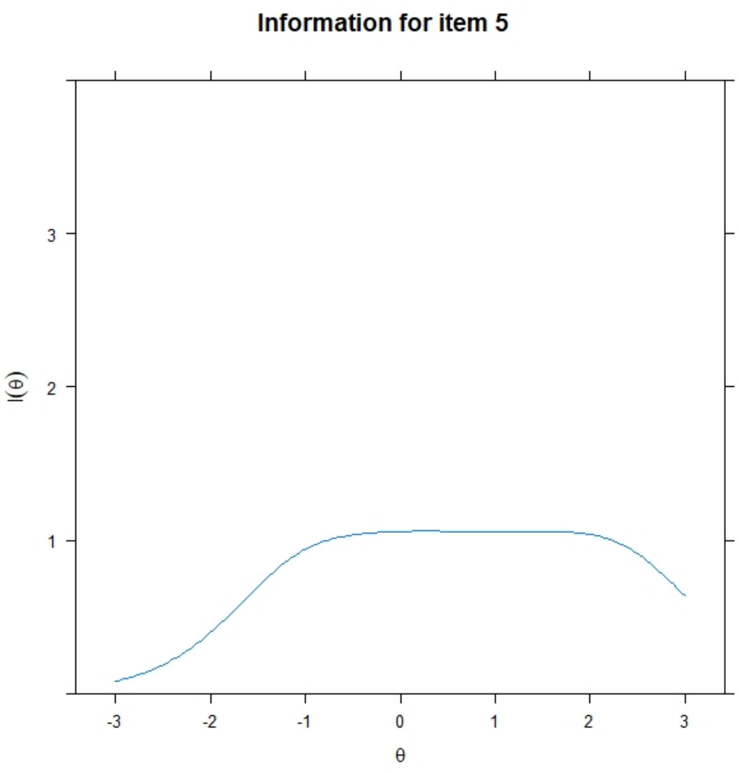

Figure 20

Item Information Curves of Thwarted Belongingness, Item 5

Note. Item 5 of thwarted belongingness=Item 20 of the INQ-25.

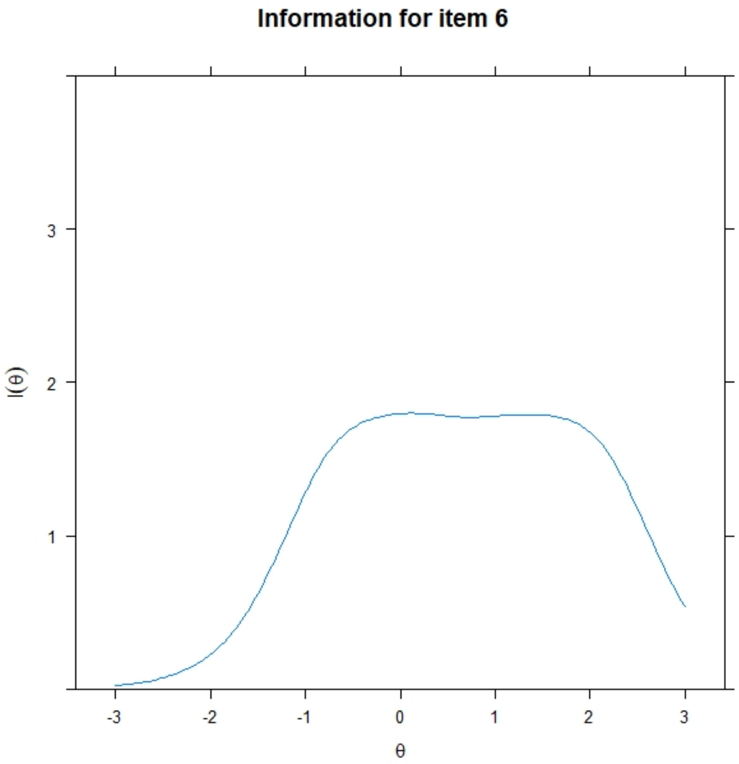

Figure 21

Item Information Curves of Thwarted Belongingness, Item 6

Note. Item 6 of thwarted belongingness=Item 21 of the INQ-25.

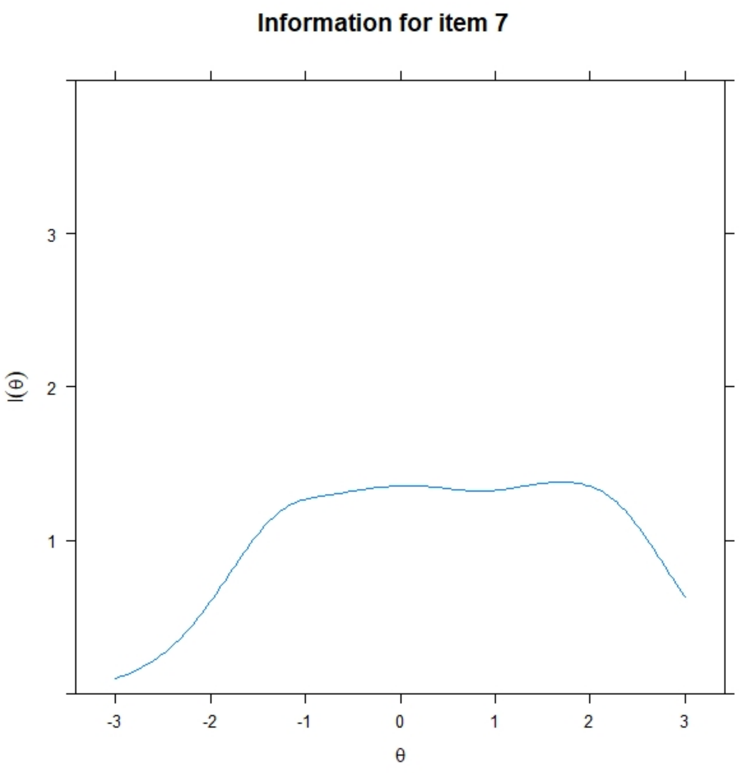

Figure 22

Item Information Curves of Thwarted Belongingness, Item 7

Note. Item 7 of thwarted belongingness=Item 21 of the INQ-25.

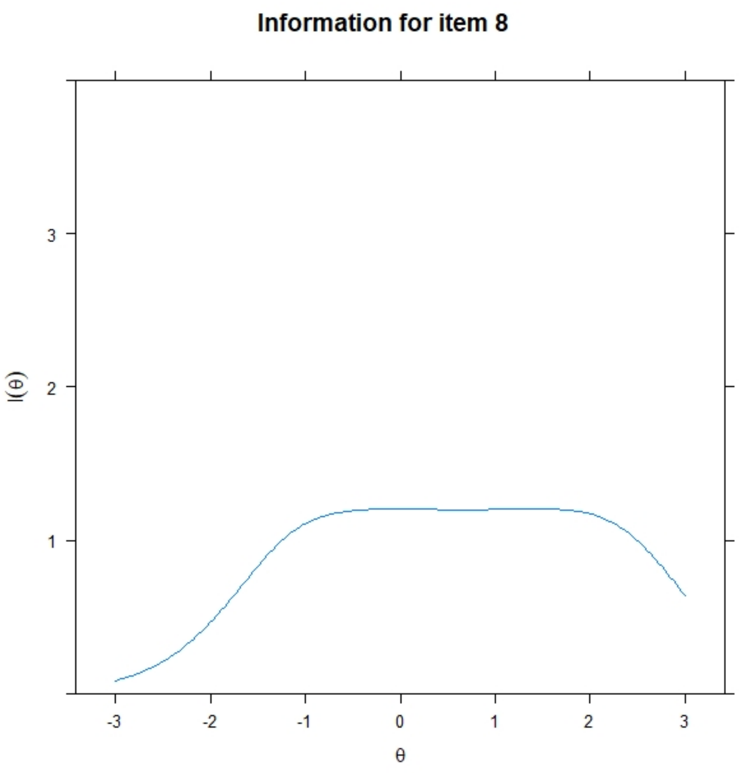

Figure 23

Item Information Curves of Thwarted Belongingness, Item 8

Note. Item 8 of thwarted belongingness=Item 23 of the INQ-25.

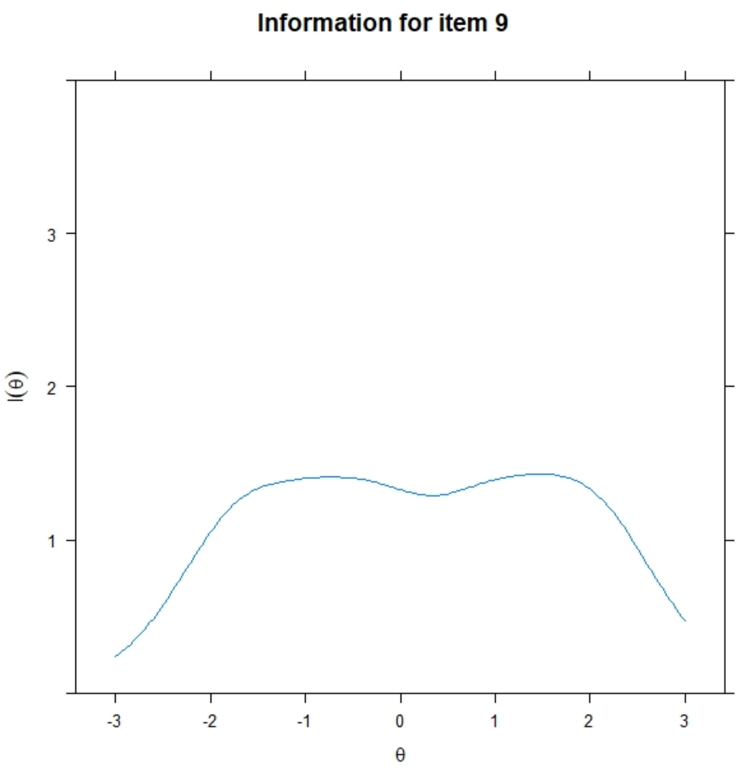

Figure 24

Item Information Curves of Thwarted Belongingness, Item 9

Note. Item 9 of thwarted belongingness=Item 24 of the INQ-25.

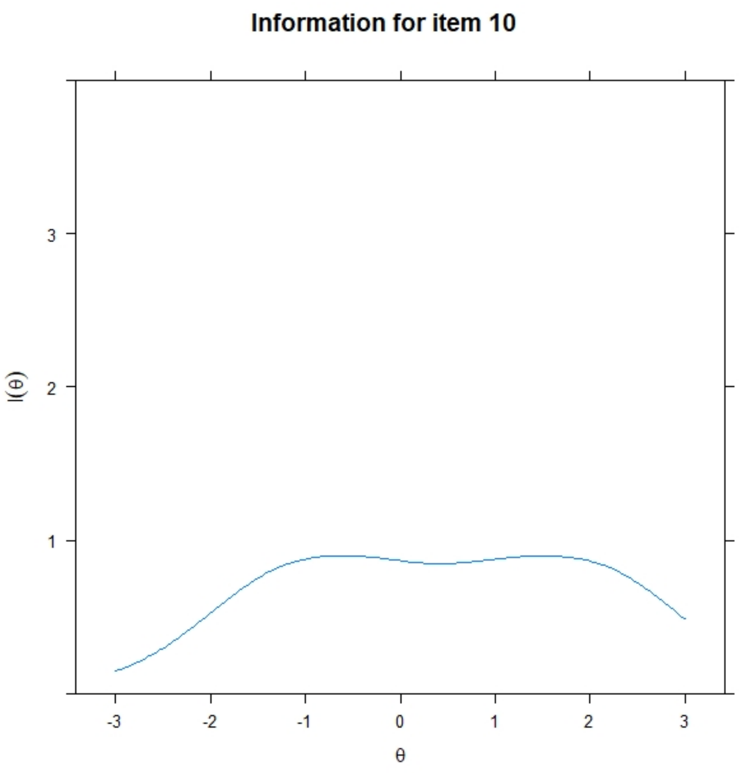

Figure 25

Item Information Curves of Thwarted Belongingness, Item 10

Note. Item 10 of thwarted belongingness=Item 25 of the INQ-25.
